# An Integrated Model of Transcription Factor Diffusion Shows the Importance of Intersegmental Transfer and Quaternary Protein Structure for Target Site Finding

**DOI:** 10.1371/journal.pone.0108575

**Published:** 2014-10-21

**Authors:** Hugo G. Schmidt, Sven Sewitz, Steven S. Andrews, Karen Lipkow

**Affiliations:** 1 Department of Biochemistry & Cambridge Systems Biology Centre, University of Cambridge, Cambridge, United Kingdom; 2 Nuclear Dynamics Programme, The Babraham Institute, Cambridge, United Kingdom; 3 Fred Hutchinson Cancer Research Center, Seattle, Washington, United States of America; Fondazione Edmund Mach, Research and Innovation Centre, Italy

## Abstract

We present a computational model of transcription factor motion that explains both the observed rapid target finding of transcription factors, and how this motion influences protein and genome structure. Using the Smoldyn software, we modelled transcription factor motion arising from a combination of unrestricted 3D diffusion in the nucleoplasm, sliding along the DNA filament, and transferring directly between filament sections by intersegmental transfer. This presents a fine-grain picture of the way in which transcription factors find their targets two orders of magnitude faster than 3D diffusion alone allows. Eukaryotic genomes contain sections of nucleosome free regions (NFRs) around the promoters; our model shows that the presence and size of these NFRs can be explained as their acting as antennas on which transcription factors slide to reach their targets. Additionally, our model shows that intersegmental transfer may have shaped the quaternary structure of transcription factors: sequence specific DNA binding proteins are unusually enriched in dimers and tetramers, perhaps because these allow intersegmental transfer, which accelerates target site finding. Finally, our model shows that a ‘hopping’ motion can emerge from 3D diffusion on small scales. This explains the apparently long sliding lengths that have been observed for some DNA binding proteins observed *in vitro*. Together, these results suggest that transcription factor diffusion dynamics help drive the evolution of protein and genome structure.

## Introduction

Control of gene regulation and cellular development relies on the ability of transcription factors (TFs), a subset of the sequence-specific DNA binding proteins (ssDBP), to activate or repress selected genes in response to internal cues or changes in the environment. To perform their function, TFs must first reach relatively small regulatory sequences within much larger genomes.

Eukaryotic chromosomes are hierarchical macro-structures of DNA and proteins, of which the DNA ranges in length from hundreds of kilobases to multiple gigabases. The basic unit is the nucleosome, in which 147 bp of DNA wraps nearly twice around the protein core of a histone octamer [1]. The resulting chromatin is further compacted into higher order structures [2]. These compact structures exist in parallel with more open domains, which have highly variable structures and topologies. Recent DNA-DNA contact maps show that chromatin is segregated into territories, in which DNA loci mainly contact regions on the same chromosome. Examples of such organisation are a fractal globular arrangement [3] and multiple solenoidal structures [2] within the nucleus, depending on the species.

Transcription factors and many other proteins interact with DNA. Their sequence-specific interactions are mediated primarily by hydrogen bonds and van der Waals interactions, while their non-specific interactions are largely based on electrostatic forces [4]. In the latter case, the negatively charged DNA filament creates an electrostatic field that attracts positively charged patches on the proteins. The radius over which the electrostatic field is effective is reduced by positive ions in the nucleoplasm, which counteract the effect of the DNA's negative charge. The resulting “Manning radius,” which is effectively the Debye length for protein-DNA interactions, typically extends 1–2 nm from the surface of a DNA filament [5], [6]. Non-specifically bound proteins are attracted weakly enough that they can typically slide reasonably freely along the DNA filament.

**Figure 1:**
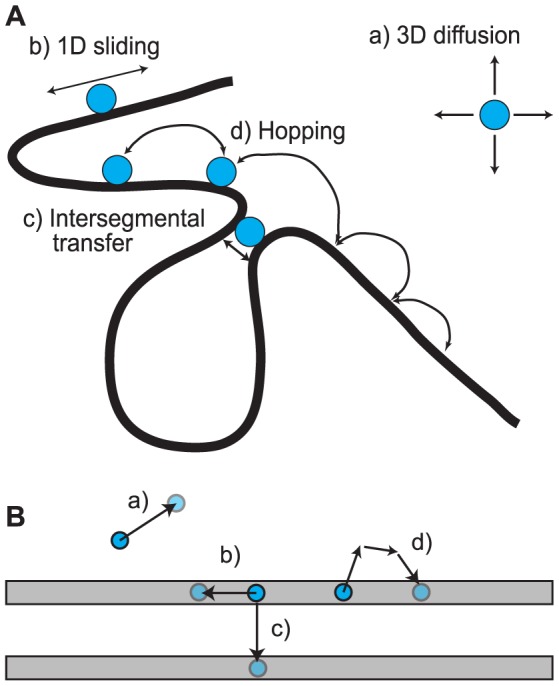
Modes of transcription factor motion. A) Schematic of the four modes of transcription factor (TF) motion (modified from [117]). B) Schematic of their implementation in the Smoldyn model. Modes: a) 3D diffusion within solution by Brownian motion, b) 1D sliding of a TF non-specifically bound to DNA, c) intersegmental transfer, where a TF binds two DNA segments and moves from one to the other, and d) hopping, in which a TF makes short excursions away from DNA (simulated as a sequence of elementary unbinding, diffusion, and binding processes).

Transcription factor motion, which takes place within this complex nuclear environment, has been investigated for several decades. Seminal work by Berg *et al.* [7]–[9] defined the four basic forms of transcription factor motion in the presence of DNA, which are collectively known as ‘facilitated diffusion’ (Figure 1; see [13], [42] for reviews). These are: (a) 3D Brownian diffusion in the nucleoplasm, (b) 1D sliding along the DNA, facilitated by non-specific TF-DNA binding, (c) intersegmental transfer (IST), where proteins transfer directly between two DNA segments that are in close proximity to each other, and (d) hopping, in which a TF unbinds from a DNA segment, diffuses briefly in 3D space, and rebinds to a nearby section of the same DNA segment. An additional form of motion is (e) intersegmental jumping, in which a TF unbinds from a DNA segment, diffuses briefly in 3D space, and rebinds to a different DNA segment nearby [10]–[12]. An important criterion of all of these search mechanisms is whether they are distance dependent or independent. This refers to the time a TF takes to find its target gene (TG), called the finding time, in relation to the distance of the TF to the TG. A mechanism is called distance dependent if the mean finding time is a function of the distance between TF and target gene, and distance independent if there is no correlation between the distance and the finding time.

3D diffusion is effectively distance independent within the bounded system of the nucleus, and so is referred to distance independent throughout this paper. It is typically the slowest of the above mechanisms [7]. However, it allows access to all of the nucleus and genome [7] [14], [15].

1D sliding is distance dependent with the finding time scaling with the square of the distance [16], [17]. This implies that target finding is rapid over short distances but very slow for long distances [16]. 1D sliding only allows searching on uninterrupted stretches of DNA. Chen *et al.* (2014) recently imaged both 3D diffusion and 1D sliding in single, living cells [18].

Intersegmental transfer finding times do not obey a simple scaling law but depend on DNA conformation and concentration [19], [20]. IST is generally an extremely rapid form of searching, as it effectively converts the DNA structure into a diffusion lattice. IST is a prevalent and important form of TF motion. For example, experimental work by Elf *et al.* [21] showed that finding times increased if IST was abrogated. This was consistent with experiments that selectively allowed IST by altering DNA conformation [11], [12] and with the observation that TFs spend a high fraction of their time bound to DNA, as opposed to being in solution [22] [21]. The importance of IST was further emphasized by recent modelling work by Bauer *et al.* [23], by fluorescent analytic work by Esadze and Iwahara [22], and by mathematical and experimental investigation of 1D sliding and IST of the Egr-1 protein [22]. Recent advances in microscopy have enabled direct imaging of IST [10], [24].

The domain structure of chromatin in a subregion of the genome affects the way in which IST may operate. If a chromatin domain approximates a solenoidal organisation or certain fractal organisations, an individual TF will be able to move by IST only between the segment it is bound to and the two that are adjacent [25]–[28]. On the other hand, if a chromatin domain approximates a more globular structure, with multiple contacts between DNA segments from different chromosomes, the TF would be able to move between different chromosomes, thereby extending its reach. This would be particularly the case if the ‘chromatin globule’ were dynamic [3], [29], [30]. We also note that some computational models specifically avoid IST and as a result find that DNA conformation is irrelevant to TF finding acceleration [31].

Hopping arises when a TF unbinds from DNA, diffuses in 3D, and rebinds to a *nearby* site on the same DNA segment, where this length scale is set by the definition that hopping is a distance dependent mechanism [13]. This contrasts with the distance independence of 3D diffusion with delayed rebinding, in which the rebinding location is essentially uncorrelated with the location where the TF unbound [13]. The diffusion length that marks the difference between 3D diffusion and hopping is not precisely defined [16] but generally accepted to be shorter than the DNA persistence length [16], [32] (about 50 nm or 150 bp). We further distinguish hopping from the situation when a protein simply returns to the DNA because it is still electrostatically attracted to it (i.e. it does not leave the Manning radius). The distance dependence of hopping is close to that of 1D sliding, occurring with an appreciable rate only over very short distances. Hopping has been difficult to verify experimentally. However, its existence is supported by recent NMR work on the HOXD9 domain [33] and by work on the Oct1 protein [34]. Hopping has also been suggested to explain the observations that interlinked plasmids are more readily cleaved [12], and that supercoiling increases restriction enzyme motion on DNA [11]. On the other hand, some recent evidence suggests that these may be incorrectly classified cases of IST [35].

Intersegmental jumping is similar to hopping, but is between separate DNA segments rather than DNA regions on the same segment [10]–[12]. We define a segment as a stretch of continuous DNA on which uninterrupted 1D sliding is possible. Intersegmental jumping is also similar to IST, with the difference that intersegmental jumping requires 3D diffusion whereas IST relies on the TF binding to two DNA segments simultaneously. However, it has proven difficult to distinguish these experimentally. In particular, the *Eco*RI protein has only one DNA binding site, and therefore should not be able to perform IST, so was assumed to move by intersegmental jumping. However, it has recently been found that its transfer kinetics are closer to those of IST. This may be facilitated by a hitherto unrecognized positive patch on the protein surface opposite to the DNA binding site of the *Eco*RI protein, which may provide the necessary structure for IST [35]. This suggests that some of the other previously observed intersegmental jumps may in fact be intersegmental transfers.

In 1970, Riggs and colleagues laid the foundations of this field by investigating the binding kinetics in the *lac* operon, finding that transcription factors locate their target genes (TGs) nearly two orders of magnitude faster than the maximum speed allowed by 3D diffusion alone (7×10^9^ M^−1^ s^−1^ versus 1×10^8^ M^−1^ s^−1^ respectively) [36], [37]. These observations have been substantiated more recently [38] and have attracted much interest. However, they come with the caveat that the fastest finding time results arose from experiments in which the salt concentration was significantly lower than in physiological conditions, which would have effectively confined transcription factor motion to 1D sliding and intersegmental transfer, thus completely avoiding slow 3D diffusion [39] [16]. This is because low salt concentrations increase the Manning radius, which effectively prevent the TF from unbinding.

These results and others showed that facilitated diffusion increases the speed of TF target finding above that of 3D diffusion alone [36], [37], [40]–[47]. See [32] [42] [48] for excellent reviews of the field, and [49] for an emphasis on computational methods of analysis. Furthermore, experimental and theoretical work are in agreement that 1D sliding is essential to faster transcription factor finding [16], [50], [51]. One of the principal conceptual problems that has emerged from this work is the speed-stability paradox for ssDBPs. It states that these proteins must bind DNA sufficiently weakly to allow for sliding but also sufficiently tightly to produce the stability required to drive gene activation, which are mutually contradictory requirements [31] [23], as it implies that these proteins must bind DNA with both weak and tight modes.

**Figure 2:**
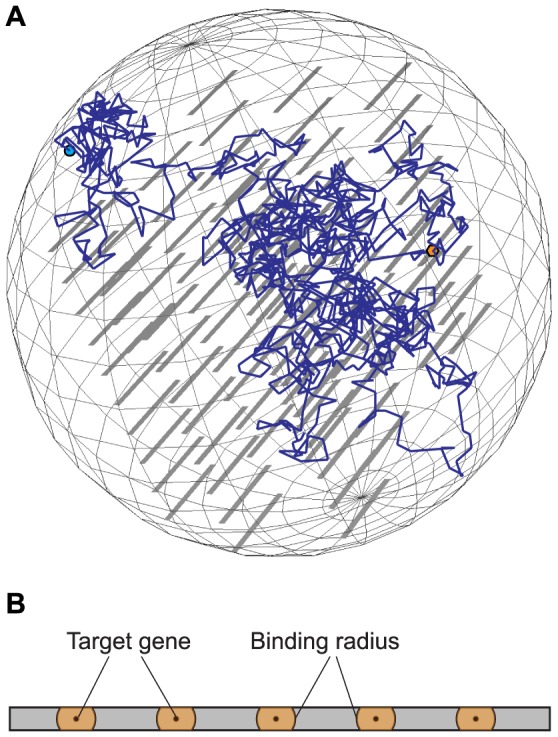
Smoldyn model of the yeast nucleus. A) Example of a Smoldyn simulation. The nuclear envelope is modelled as a perfect sphere containing one or more TFs (light blue dot), multiple stacks of DNA segments (grey bars), and one or more target genes (TG) on the DNA (orange hexagon). Individual DNA segments may be part of the same chromosome, but are separated so that 1D sliding is not possible between them. B) Target genes along a DNA filament, shown with their centres as black dots and their binding radii as orange regions. TF-TG complex formation occurs when a DNA-bound TF diffuses to within a binding radius of a TG.

Here we present a computational model of TF motion (Figure 2). It allowed us to analyse the contributions of the individual TF motion components and enables us to explain the large increase in speed of TF-TG finding first reported by Riggs *et al.* We simulated TF movements using the computer program Smoldyn, previously used for modelling signal transduction and related phenomena within or between individual cells [52]–[56]. Building on the established observation that 1D sliding leads to shorter finding times, we find that 1D sliding has an upper maximum search distance determined by diffusion dynamics. This maximum turns out to be a good predictor of both empirically observed TF-DNA unbinding constants and a good fit to the length of nucleosome free regions (NFRs) in eukaryotes. Our work further confirms the importance of IST and also shows how IST can lead to two forms of searching. One is distance independent and most likely to occur in areas of high concentration of chromatin, while the other is distance dependent and most likely to occur in areas of low chromatin concentration. From published data, we found that TFs and other sequence specific DNA binding proteins are enriched in dimers and tetramers, both of which promote IST.

## Model

### Model description

We based our model of TF motion on the yeast nucleus. The aspects of yeast nuclei that are relevant to this work are similar to those of other eukaryotes, so our model will equally apply to sequence-specific protein-DNA interactions more generally, and not just to those in yeast. Our model consists of a 1.5 µm diameter sphere, modelled on the size of the yeast nucleus [57], which contains one or more stacks of virtual DNA segments. These DNA segments are 2.6 nm wide and vary from 10 to 4,200 bp in length, depending on the specific analysis. These are much shorter than yeast chromosomes (diploid cells have 32 chromosomes, ranging from 200 kb to 1.5 Mb) but, from the point of view of TF searching by 1D sliding, they are taken to be appropriate because chromosome lengths that are accessible to sliding are limited by heterochromatinisation and other obstructions. Also for this reason, we only included stretches of DNA that are accessible to the diffusing TF and are not heterochromatic or otherwise occluded. This is analogous to the way that *in vitro* studies of ssDBPs interacting with masses of discontinuous DNA have been argued to provide results that are generalisable to *in vivo* ssDBP-genome interactions [33]–[35]. We modelled these DNA sections as long narrow rectangles with each rectangle side representing a DNA groove. Rectangle widths were 2.6 nm, corresponding to the DNA double helix width [58].

We modelled TFs as dimensionless points. They diffused on DNA surfaces with a 1D diffusion coefficient of 0.0262 µm^2^/s, which was based on our analysis of fluorescence recovery after photobleaching (FRAP) data for the Ace1p yeast transcriptional activator from [59] and was comparable to other measurements [21], [38], [45], [60]–[64]. We simulated this as 2D diffusion, but the long narrow DNA rectangles made it effectively 1D. TFs did not diffuse off the ends of DNA segments, but instead reflected back towards where they came from. When dissociated from DNA, TFs diffused within the 3D nuclear volume with a diffusion coefficient of 2.72 µm^2^/s. This is 104 times faster than 1D diffusion, which we based on theoretical work by Berg *et al.* (1982) [8] and experimental work on bacterial transcription factor diffusion [45], [63]–[65]. Our assumed 3D diffusion coefficient is comparable to that for nuclear FITC-dextrans [14].

TFs bound non-specifically to the two DNA rectangle faces, which acted as uniform binding areas, as adsorption processes. We varied the adsorption coefficient, *k_on_*, from 1.7 µm/s for chromatinised DNA to 10 µm/s for protein-free DNA. TFs dissociated from these non-specifically bound states at a rate, *k_off_*, of 11.6 s^−1^, which we again based on the FRAP measurements of Ace1p [59], verifying it by replicating the recovery curve given in that work (Figure S1, compare to Figure S5 in [59]). This dissociation rate also compares well to similar studies of transcription factor binding [41], [66].

We simulated IST as random TF transfers from one DNA segment to an adjacent one, with a rate constant of 11.6 s^−1^. We ignored the physical proximity of these segments, which accurately captured the *effect* of concentrated and organised DNA but without needing to accurately reconstruct the DNA conformation (cf. [44]; and [23]). Because hopping occurs on a size scale below that of the DNA persistence length [16], [32], our use of straight DNA segments in the model had minimal impact on hopping motions.

We represented each TF-specific target gene (TG) as a point on the centreline of the DNA rectangles. TFs diffusing in 3D space did not interact with TGs (with some exceptions, noted below). On the other hand, TFs that were already non-specifically bound to DNA could bind to TGs, which happened when a TF diffused to within one “binding radius” of a TG. We used a binding radius, *σ_b_*, of 2.0 nm (about 6 bp), which Smoldyn computed using our assumption of a TF-TG binding rate constant of 10^5^ M^−1^ s^−1^ (comparable to ssDBP binding measurements [67]–[77]). This binding radius is comparable to the size of recognition sequences. More importantly though, it is substantially wider than the 1.3 nm DNA half-width; this meant that when a DNA-bound TFs diffused towards a TG, it nearly always bound the TG and did not diffuse around it (the TF diffused in discrete Gaussian-distributed displacements with 2.3 nm rms step lengths, so it was possible but unlikely for a TF to step completely over a TG). For this reason, the values used for the TF-TG binding rate constant had essentially no effect on our simulations. We modelled TF binding as a change of species, converting it from a rapidly diffusing TF to a bound TF-TG complex. This is much like the change from the fast ‘search mode’ to the immobile ‘recognition mode’ of the speed/stability paradox [48]. In some simulations, we also enabled TF-TG dissociation. In these cases, TF dissociated directly to 3D space at a rate of 0.025 s^−1^, once again based on the FRAP measurements of Ace1p [59]. All simulation parameters are summarised in Supplementary Table S1.

We did not explicitly represent heterochromatinised DNA, chromosomal packaging material, RNAs, or other proteins in addition to the modelled TFs, all of which create macromolecular crowding effects in the cell [78]. This was because the values for the parameters we used in our simulation were taken from experimental measurements. These *in vivo* values already account for any crowding effects, representing the diffusion characteristics of proteins within the crowded, intranuclear environment. Additionally, preliminary work showed that simulating crowding with up to 30% volume exclusion with 100 impenetrable spheres did not affect results significantly [79]. This agreed with our prior modelling work, with similar temporal and spatial scales, where we found that the primary effect of macromolecular crowding was to reduce diffusion coefficients [80]. It also agreed with fluorescence imaging results which showed that protein motion on the scale of transcription activators is not significantly affected by chromatin or other crowding agents [15] and is in accord with the observed high diffusion rates within the nucleus [14], [21].

### Model simulation and validation

We simulated our model in Smoldyn (versions 2.09 to 2.31), which is a particle-based simulator of diffusion, reactions, and surface interactions [53]. Whereas many mathematical models of cell biology are deterministic, specifying constant rates for processes (e.g. specifying the number of times hopping occurs in a given unit of time [44], [81]), Smoldyn accounts for stochastic behaviour accurately, which means that complex processes emerge naturally from the fundamental diffusion dynamics. Unless otherwise specified, we ran each simulation for 1 virtual hour and used time steps of 0.1 ms. This time step caused the simulation spatial accuracy to be about 7 nm for TFs in 3D and 0.07 nm for TFs bound to DNA. This detail was fine enough to capture hopping and other TF motions, but also coarse enough, in contrast to simulators with single DNA basepair resolution such as GRiP [49], that we were able to run simulations quickly. We ran single simulations using a Mac Pro computer (2×2.8 GHz quad-core Intel Xeon), which typically completed in seconds to minutes, and batches of hundreds of simulations overnight using the Cambridge University computing grid. We analysed Smoldyn output using MATLAB version 2013a. Smoldyn input files, MATLAB scripts and simulation parameters are included as Supplementary Information.

We validated our simulations in several ways. First, we found in prior work that Smoldyn's simulations of diffusion, binding and unbinding reactions, and surface reactions all have kinetics that differ from theoretical predictions by less than 2.5% [53], [82], [83]. Secondly, we tested for expected behaviours and robustness as we varied simulation parameters (see [79]). As expected, simulations that investigated just reversible TF-TG complex formation, without virtual DNA, showed that the final number of TF-TG complexes increased nearly linearly with both TF and TG counts (not shown). Also, varying 3D diffusion coefficients showed few final complexes with 10^5^ fold slower diffusion than our typical assumption due to slow equilibration, and the equilibrium number of complexes when diffusion coefficients were 0.027 µm^2^ s^−1^ or greater, again as expected. Varying TG locations and the extent of TG clustering showed that these had minimal effects on results. Finally, we tested the distance dependence of finding times in different dimensionalities. We placed a cluster of 5 TGs (a) onto a long narrow rectangle that represented DNA to test 1D finding times, (b) onto a square flat surface to test 2D finding times, or (c) freely into space within the 1.5 µm diameter nuclear envelope to test 3D finding times; the outer dimensions of each system was 2 µm long on each axis. Then, we released 5 diffusing TFs a fixed distance away from the 5 TGs and recorded the mean finding time (Figure S2). As expected, the average finding time increased continuously with the distance for the 1D and 2D cases, but was essentially distance-independent for the 3D case. Together, these tests suggested that our simulations worked as intended.

## Results

### 1D Sliding has a limited range that is only moderately influenced by the unbinding constant

**Figure 3:**
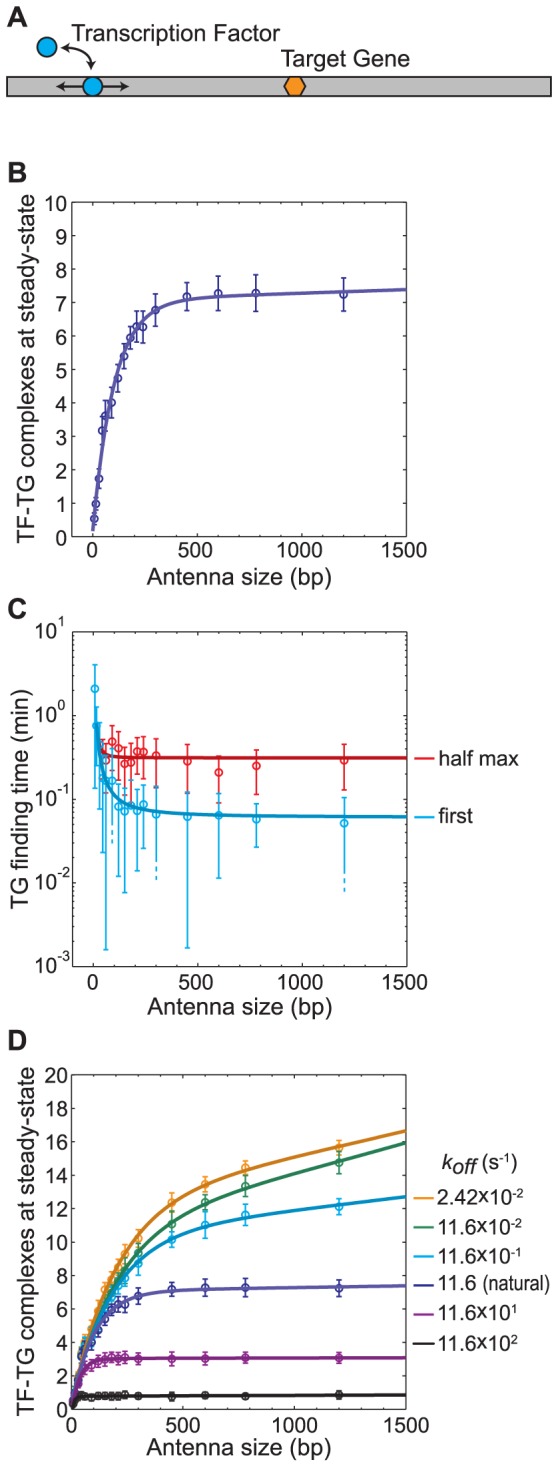
Antenna effect. A) Illustration of the ‘antenna effect’: target gene finding times are reduced when TFs can get to their targets by diffusing along the DNA. The TF (light blue circle) diffuses along the antenna DNA (grey bar) to reach the TG (orange hexagon). B) Effect of antenna length on the number of TF-TG complexes at steady-state. C) Effect of antenna length on the time for the first TF to bind to the first TG in the simulation (blue) and on the time required for half of the steady-state number of TF-TG complexes (from panel B) to form (red). D) Effect of the DNA dissociation rate (*k_off_*) and antenna length on the number of steady-state TF-TG complexes. Simulation parameters: *D*_3D_ = 2.72 µm^2^ s^−1^, *D*_1D_ = 0.0262 µm^2^ s^−1^, *k_on_* = 1.7 µm/s, *k_off_* = 11.6 s^−1^ unless otherwise noted, *σ_b_* = 2 nm, IST rate = 0, and specific binding was reversible with dissociation rate 0.025 s^−1^; 50 TFs were started at random 3D locations and there were 20 TGs, each at the centre of a DNA segment. Error bars represent one standard deviation, determined from 20 repeated simulations.

The ‘antenna effect’ is defined as the process by which ssDBPs find their targets more readily if the recognition sequence is embedded in nonspecific DNA on which 1D sliding (and/or hopping) is possible [84]–[86]. We investigated this effect by placing 20 TGs at the centres of 20 DNA segments and starting 50 TFs at random locations in the 3D nuclear volume (Figure 3A). We ran each simulation 20 times, and counted the number of steady state complexes formed. They confirmed the antenna effect: the steady state number of complexes was a function of the antenna length (total DNA segment length), increasing sharply up to about 300 bp and more gradually for longer antennas (Figure 3B). Longer antennas conferred minimal additional advantage toward forming TG-TF complexes, which arose from the increasing likelihood of TF dissociation from the DNA. Presumably, the number of steady-state complexes would have turned around and started decreasing at some point if we had investigated extremely long antennas, due to TF sequestration, but these effects did not appear for the DNA lengths that we used.

Figure 3C shows that the target finding time was also a function of the antenna size, both for the first target bound (cyan line) and for the half-maximal number of targets to bind (red line, half-maximal values were computed from the steady state values plotted in panel B). In both cases, finding times decreased sharply for antenna lengths up to about 300 bp and hardly at all for longer lengths. Again, short antennas were highly effective but longer ones conferred minimal additional advantages.

Because the effectiveness of long antennas is largely limited by TF dissociation from the DNA, we explored the effect of varying the TF-DNA dissociation constant (*k_off_*) on the number of TF-TG complexes at steady-state (Figure 3D). As expected, faster dissociation resulted in both fewer complexes and shorter lengths over which antennas were effective. At the other extreme, the dissociation rate had minimal effect on the number of complexes when it was very slow (compare upper curves of Figure 3D). In these cases, TFs typically did not dissociate from the DNA until after forming TF-TG complexes. We thus find that antennas do not gain substantial effectiveness for lengths that are greater than about 300 bp or for *k_off_* rates that are below about 1 s^−1^.

### Hopping on ‘naked’ DNA as an emergent property

**Figure 4:**
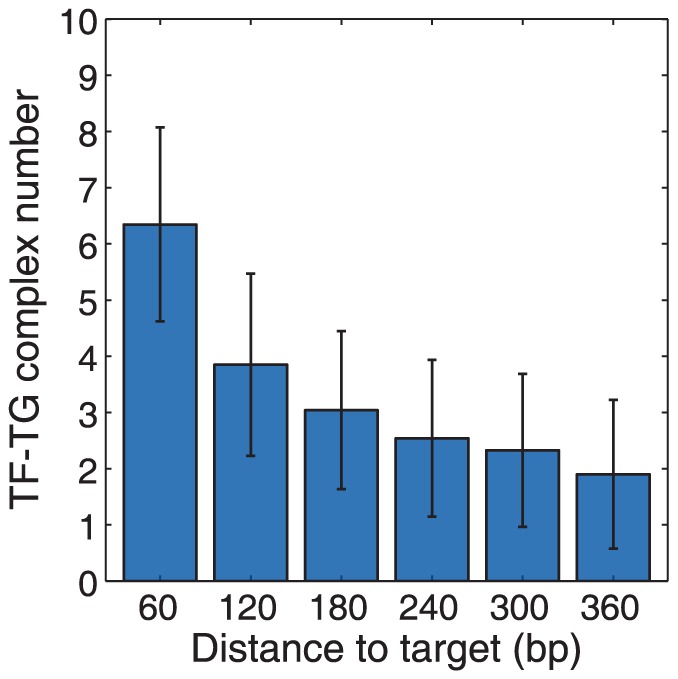
Transcription factor hopping. The number of TF-TG complexes that formed for TFs that started at various distances away from their targets and that could not slide along the DNA. The distance dependence shown here is indicative of hopping motion, in which TFs repeatedly unbound from the DNA, diffused briefly in 3D space, and rebound to the DNA at a location close to the unbinding location. Simulation parameters: *D*_3D_ = 2.72 µm^2^ s^−1^, *D*_1D_ = 0, *k_on_* = 1.7 µm/s, *k_off_* = 11.6 s^−1^, *σ_b_* = 2 nm, IST rate = 0, specific binding was irreversible, and multiple TFs binding to a single TG was allowed; on each of 20 DNA segments, 6 labeled TFs were started at 60 bp distance increments away from a single TG. Bar heights represent the number of TF-TG binding events, out of 20 possible, for each TF location after 60 minutes. Error bars represent one standard deviation, determined from 20 replicate simulations.

The hopping mode of ssDBP motion can be difficult to identify because of its short range. Because TF diffusion is not biased to move towards a particular DNA end, hopping is equally likely to carry the TF in either direction along the filament. Thus, the mean displacement over time of hopping is 0 bp. However, by measuring distance as an absolute value, the average ‘hop’ has been estimated to cover between one and several bp [44], [87]–[90]. We simulated TF hopping by placing 6 individually identifiable TFs at varying distances from a single TG on each of 20 DNA segments, each 930 bp long. We allowed multiple TFs to bind to each TG and used the individual TF labels to determine which ones bound. We prevented TF-TG complex dissociation and 1D sliding, but allowed TF-DNA unbinding and rebinding. At the end of the 60 minute simulation, we counted the number of TF-TG complexes for each of the TF labels (Figure 4). Using our standard simulation parameters, including a a DNA adsorption coefficient of 1.7 µm/s, the number of complexes formed was independent of the initial TF distance away from TGs (not shown). This indicated that hopping did not occur appreciably in this case, but that TF-TG complex formation occurred via distance independent 3D diffusion. However, increasing the DNA adsorption coefficient to 10 µm/s produced complex counts that did depend on the distances (Figure 4), thus implying the presence of hopping [7], [48]. The necessity of this larger adsorption coefficient suggests that hopping may only occur to an appreciable extent on “naked” DNA, meaning DNA that is not bound to histones or other obstructions. Additionally, Figure 4 shows that hopping can be effective on distances of 360 bp or more, but its effectiveness drops off rapidly after about 60 bp. Conceptually, hopping increases the effective range of 1D sliding.

### Intersegmental transfer provides two modes of motion

**Figure 5:**
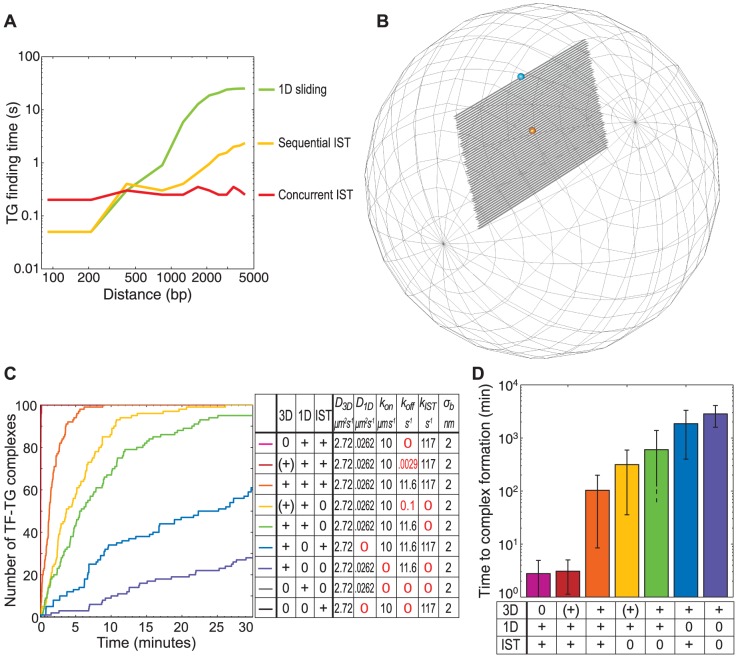
Intersegmental transfer. A) Mean finding time for a single transcription factor placed at varying distances away from its target. Searching was possible by 1D sliding (green), “sequential” intersegmental transfer between adjacent DNA sections (yellow) or “concurrent” intersegmental transfer between all DNA sections in a group (red). B–D) Simulations testing the effect of the three major modes of motion. B) Arrangement of the 50 DNA segments within the simulated nucleus: shown 10× wider than in the simulation for clarity. The TF is placed at the position shown in light blue, and the time is measured until it binds the target gene (orange sphere). C) Total number of complexes formed over time for 100 different simulations, each with one transcription factor and one target, using nine different combinations of 3D diffusion, 1D sliding, and intersegmental transfer (IST). 3D diffusion was varied between low (+), standard +, and zero, 0. 1D sliding and IST was either present, +, or absent, 0. Shown adjacent are the corresponding values for the simulation parameters: *σ_b_* is the binding radius for TFs in 3D space and also bound to DNA. 1D sliding or IST alone (grey or black line) did not achieve a single binding event in the time shown. D) Mean target finding time, in the same simulations as shown in C).

We investigated the effect that varying forms of chromatin organisation have on IST with three sets of simulations. The first set did not include IST; here, we placed a TG at one end of a 4,200 bp DNA segment, started a TF at a fixed distance away from the TG, and restricted TF motion to 1D sliding. The second set used “sequential IST,” which might occur in solenoidal DNA structures or in regions of low chromatin concentration. Here, we created a stack of ten DNA segments, each 420 bp in length, placed a TG at one end of the top segment, and started a TF at a fixed position on one of the other segments. We restricted TF motion to 1D sliding and IST between adjacent segments. The third set used “concurrent IST,” which might occur in a fractal globular DNA structure. We simulated this in the same way as for the sequential IST, but allowed IST between all segments. In the latter two cases, the plotted distance is the total DNA length between the TF and the TG. As expected, finding times were strongly distance dependent for 1D sliding (Figure 5A). On the other hand, they were less strongly distance dependent for sequential IST and essentially distance independent for concurrent IST. These results agree with prior work that has shown that IST generally accelerates finding times [11]. They also emphasize the point that the effect of IST depends strongly on the local chromatin concentration and structure [19], [20].

### Combining intersegmental transfer and 1D diffusion accelerates TF-TG finding

We investigated the relative effectiveness of the different types of TF motion using simulations that included them in various combinations and with various rate constants. These simulations allowed one or more of 1D sliding, 3D diffusion, and IST, and varied the *koff* rate constants. We did not specifically prevent hopping, so this undoubtedly occurred whenever 3D diffusion could. In all cases, our simulations included a stack of 50 DNA segments of 1.5 kb each, for a total of 75 kb (Figure 5B). The end of each segment linked to the start of the next, such that a TF could slide from one segment to the next. Each simulation included a single TF that started at one end of the DNA stack and a single TG that was located at the opposite end of the stack. Simulations treated TF-TG binding irreversibly and ran for 2 hours. Whereas we typically only allowed TF-TG complex formation for TFs that had already bound to the DNA, we relaxed that requirement here, also allowing TFs in 3D space to bind to TGs, using the same 2 nm binding radius. We repeated each simulation 100 times.

Figures 5C and 5D show the final number of complexes from the 100 simulations and their average finding times. Simulations that only allowed 1D sliding produced no complexes within 30 minutes because 1D sliding is very slow over long distances. IST alone was also completely ineffective, in this case because the TF couldn't search along the DNA, but was restricted to the relatively few sites where it randomly landed. 3D diffusion alone did produce complexes, but relatively slowly. In this case, a complex only formed if the TF diffused into the 2 nm binding radius of the TG, by 3D diffusion, which made this an infrequent occurrence. Combining 3D diffusion with either 1D sliding or IST alone accelerated the complex formation rate by less than a factor of 10. On the other hand, when 1D sliding and IST were combined together, then complexes were formed several orders of magnitude faster. This combination was most effective when the TF could not diffuse significantly in 3D space, and thus stayed confined to the DNA filament (Figure 5D, compare first two bars with the third bar).

Notably, when we used our default parameters for all types of motion, which we estimated as well as possible from experimental data (shown in orange, [+ + +] in Figure 5C and 5D), the finding time fell in the range of 2–3 minutes, similar to that observed by FRAP measurements [59]. This finding time is faster than we found in simulations that only combined 1D and 3D diffusion (including hopping). This suggests that IST plays a strong role *in vivo* and is essential for explaining experimentally measured finding times.

### Transcription regulators are enriched for dimeric and tetrameric structures

Based on observations by us and others that IST acts as a strong accelerator of TF-TG complex formation, we wondered whether this would be reflected in the way that TF structures have been shaped by evolution. This would be reasonable because the time for a TF to find its recognition sequence has been shown to be the rate-limiting step in transcription [91], so it should be subject to selection. In particular, we investigated whether TFs and other ssDBPs are more likely to form dimers and tetramers than other proteins. This is based on the logic that dimers and tetramers allow two DNA segments to be bound simultaneously, which is the essential requirement for IST [9], [92], as seen with the *lac* repressor and other TFs [93]. Additionally, transcription activator multimerisation has been shown to aid IST [21]. On the other hand though, ssDBPs may also use unstructured protein tails for IST [19], [20], [39], showing that IST can also happen without multimerisation [35], [94].

We used the 3D protein complex database produced by Levy *et al*. (2006) [95], who curated the set of structures deposited in the RCSB protein databank (www.rcsb.org, [96]), for a non-redundant set of protein structures. From this dataset, we counted the numbers of dimers and tetramers in the classes listed as *transcription*, *DNA binding*, *transferase*, and *oxidoreductase*, as well as in the complete curated set. The protein databases might be biased towards multimers and homodimers for technical reasons of crystallisation, but this bias should apply equally across all protein families. We are hence basing our analysis on the comparison of percentages between different protein families.

**Figure 6:**
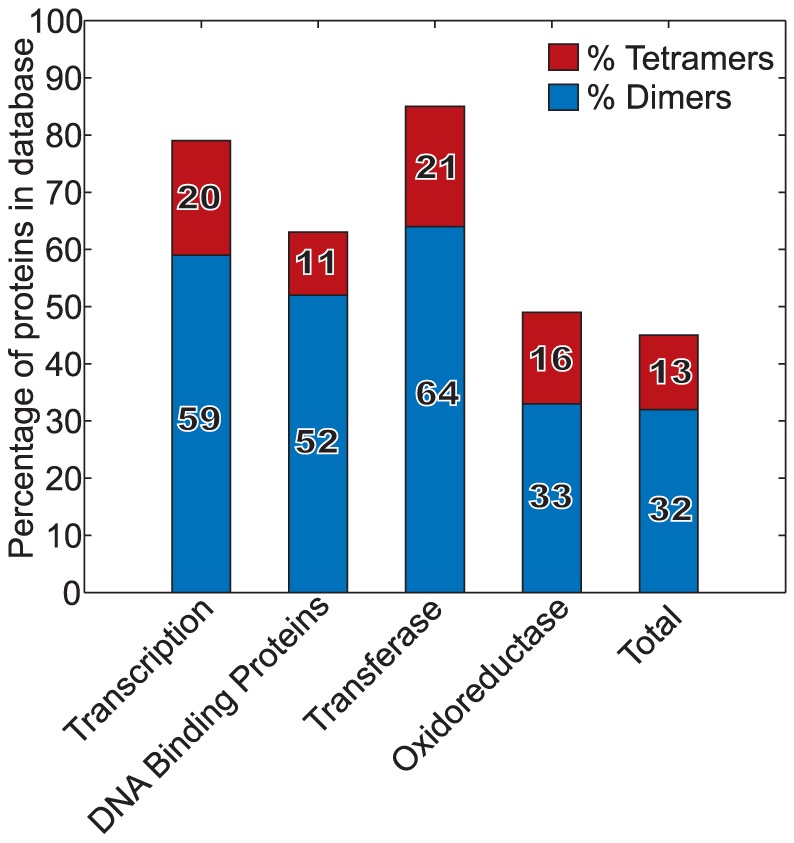
Multimericity of protein structures by PDB class. Bars show the percentage of proteins in different protein classes that form dimers (blue) and tetramers (red). The protein list, which is non-redundant, was obtained from the 3D complex database [118]. The first three bars represent sequence specific DNA binding proteins, the oxidoreductase bar is a control group that does not bind to DNA, and the final bar represents all proteins in the database.

Figure 6 shows these values, as percentages, by class. The *total* column shows that the complete list of curated protein structures comprises ca. 32% dimers, ca. 12% tetramers, and ca. 55% others (mostly monomers). The *oxidoreductase* column represents our control class because these enzymes, which are widely used in metabolism, typically do not bind to DNA. They exhibit essentially the same fractions of dimers and tetramers as the total class. The three other classes, all of which represent sequence specific DNA binding proteins, have substantially higher fractions of dimers and especially tetramers. In particular, transcription factors, the focus of this work, are 79% dimeric or tetrameric, compared to ca. 45% in the set of all proteins. Transferases also play central roles in gene regulation, including for example methyl- and acetyltransferases that perform sequence specific DNA or histone modification; they are 85% dimeric or tetrameric, with the highest percentages for tetramers of the classes analysed. IST has been observed for this class of regulators [16], [97]–[99]. Finally, the class of other DNA binding proteins is 63% dimeric or tetrameric. Thus, all three classes of sequence specific DNA binding proteins exhibit strong enrichment for dimers and tetramers. This is consistent with the possibility that evolution favoured their multimerisation in order to facilitate IST and thus reduce target finding times.

## Discussion

Our model of transcription factor motion describes known and emergent methods of how TFs find their target genes. This question has been intensively studied in the past, and our model is based on extensively validated experimental data. Using Smoldyn, we show the degree to which intersegmental transfer (IST) accelerates TG finding, and that this feature has significant impact on the evolution of the quarternary structure of ssDBPs. We are also able to give an explanation for an observation that has caused some disquiet within the community, namely the fact that TFs are able to find their targets at speeds exceeding the 3D diffusion limit. In our analysis, this can be explained by the effect of limiting TFs to 1D diffusion and IST, as is the case at low salt concentrations (see details below).

### Intersegmental transfer explains high acceleration of transcription factor search in low salt conditions

Lowering salt concentrations decreases the electrostatic shielding around DNA filaments, which increases the Manning radius and hence increases non-specific binding between ssDBP and DNA. This can largely confine TFs to the DNA, making them only able to move by 1D sliding and IST [16]. Lowering salt concentrations has also been shown to increase ssDBP searching speeds [100]. However, this acceleration only occurs if IST can take place; this has been shown with *lac* repressors that have been mutated to make them unable to form tetramers and thus unable to bind two DNA segments simultaneously and undergo IST [21].

Our simulations help explain these experimental results, as well as the original *in vitro* low-salt experiments by Riggs *et al.* [36], [37]. In particular, Figures 5C and 5D show that finding times decrease by several orders of magnitude when TF motion is restricted to 1D sliding and IST. Thus, the very fast finding times observed by Riggs *et al.* can be explained by assuming that their low salt conditions restricted TF motion to 1D sliding and IST.

### The ‘antenna effect’ may determine the length of nucleosome free regions

We showed that the ‘antenna effect’ has an effective upper limit of about 300 bp. This length is largely determined by the dissociation rate of non-specific TF-DNA interactions, which sets the residence time of TF being bound to a DNA filament, and thus determines how far the TF is likely to slide. Our dissociation rate constant value, *k_off_* = 11.6 s^−1^, which we derived from FRAP measurements of nonspecific TF-DNA interactions, is comparable with dissociation rates that have been observed for several other transcription factors [66]. In further support of our *k_off_* value, it also appears to be reasonably optimal for efficient ssDBP-DNA interaction, based on our observation (Figure 3D) that larger *k_off_* values led to many fewer TF-TG complexes, but that smaller *k_off_* values did not produce substantial further increases. In addition, the 300 bp antenna effect range that we found is consistent with the finding that the restriction enzyme *Eco*RV covers a similar distance by 1D sliding on DNA before dissociation [101] (longer sliding distances have also been observed for other restriction enzymes [102] and ssDBPs [103], but we speculate that those observations may have included hopping or IST).

Nucleosome free regions (NFRs) are sections of nucleosome free DNA typically associated with gene promoters. In yeast, NFRs are typically ∼100–200 bp in length [104]–[107] and can be found at the 5′ end or the 3′ end of a gene. The 5′-NFRs contain a marked enrichment for TF binding sites [107]–[110]. Similarly, 3′-NFRs are enriched for sequences responsible for transcription termination sites (TTS) [111]. These sites are actively maintained free of nucleosomes by the action of the ATP-driven chromatin remodelers, such as Isw2 [104], [112], [113]. The activation of transcription is accompanied by the eviction of one or two nucleosomes, which in effect extends the usual NFR of to a length of 400–600 bp in these genes [114], [115]. The similarity between the lengths of NFRs and the length of DNA traversed during effective searching is striking. We suggest that NFR lengths may be actively adjusted so as to maintain the amount of free DNA that ssDBPs require to locate their sequences efficiently. Additionally, of course, active modification of NFR lengths would create a transcriptional regulatory mechanism through modulation of the antenna effect.

### Predictions made by our model

Our work enables several predictions. First, it predicts that abrogation of multiple DNA binding domains on ssDBPs should make them unable to undergo IST and thus have increased finding times. This was already shown in studies of the *lac* repressor [21]. It should also apply to other multimeric transcription regulatory proteins and to proteins that bind DNA in other ways, such as with the disordered N-terminal tails exhibited by *Hox* proteins and others [20], or the positive patches seen on the non-DNA binding side of *Eco*RI [35]. Secondly, and conversely, our model predicts that favouring IST, such as by lowering salt concentrations, should generally decrease finding times. This was our explanation for the Riggs experiments, but should also apply to other proteins that are capable of IST. Third, based on the fact that IST appears to be important for ssDBPs to locate their target sequences, we argue that most ssDBP proteins will be enriched in structures allowing for IST. Indeed, we observed a substantial enrichment for dimers and tetramers for ssDBPs. Dimerisation of TFs has other known benefits, such as being able to recognise a longer stretch of DNA, bringing with it increased specificity, which in itself has evolutionary advantages. The effects we see are expected to be additive to all other mechanisms favouring the formation of dimers and tetramers. The differences may be even more marked if positive patches and unstructured tails are analysed as well, as has already been partly shown [116].

Finally, we suggested that the size of nucleosome free regions is influenced by the upper limit of the effective antenna length. If this is the case, then shortening the NFRs around a reporter gene should substantially reduce expression but lengthening them should increase expression only minimally. Our work suggests that these effects should be reasonably independent of the sequence within the NFR, so long as the NFR remains protein free.

### Conclusions

We present a computational model of transcription factor motion within cell nuclei that is based on only a few transcription factor elementary processes: diffusion in 3D space and along DNA filaments, non-specific binding and dissociation with DNA, specific binding and dissociation with target genes, and intersegmental transfer between DNA segments. This model exhibited the main modes of transcription factor motion, which are 3D diffusion, 1D sliding, hopping, and intersegmental transfer (we did not investigate intersegmental jumping). It showed that the antenna effect, in which transcription factors find their targets more quickly if the targets are embedded in DNA on which 1D sliding is possible, is extremely effective for antenna lengths up to about 300 bp but is not improved substantially with even longer antennas. From this result, we speculated that cells maintain nucleosome free regions about genes in part to accelerate expression through the antenna effect. Our model also showed that transcription factor hopping, defined as alternating DNA binding and 3D diffusion that has a distance dependent finding time, emerged naturally from our simulations; however, it required relatively rapid DNA binding and was only effective over a short distance. Additionally, our model reiterated the importance of intersegmental transfer. It showed that intersegmental transfer is essential for efficient target finding, and that this has likely led to a substantial enrichment of dimers and tetramers for sequence specific DNA binding proteins through evolution.

## Supporting Information

Figure S1 **Reconstitution of FRAP recovery curve.** As part of our methods verification, we ensured that we could replicate the FRAP recovery studies of Karpova *et al.* (2008) [59]. This figure shows one example, a test of the unbinding constant of a TF from its TG. As in the work of Karpova *et al.* (2008), half unbinding is reached at c. 40 seconds, and full unbinding at c. 2 minutes.
(EPS)

Figure S2 **Distance dependence of finding times in 1D, 2D, and 3D systems.** The mean time for a single TF to locate and bind a single TG is shown as a function of their initial separation for 1D, 2D, and 3D systems, in (A), (B), and (C), respectively. As expected, results are strongly distance dependent in 1D, moderately distance dependent in 2D, and nearly distance independent in 3D. Simulation parameters: *D* = 2.72 µm^2^/s, *σ_b_* = 2 nm, binding was irreversible, and the system was 2 µm wide in each dimension; 5 TFs were started at fixed distances away from 5 TGs that were all located in the centre of the system. For each simulation, we computed the mean binding time for these 5 TFs. We repeated simulations 100 times each and computed the means (solid line) and standard deviations (error bars) of the mean binding times.
(EPS)

Table S1 Summary of the simulation parameters used.
(PDF)

Code S1 Archive of Smoldyn configuration files, Matlab scripts and Python code for simulations and data analysis. See enclosed README file for details.
(ZIP)
