# Supplementary material for: An Integrated Model of Transcription Factor Diffusion Shows the Importance of Intersegmental Transfer and Quaternary Protein Structure for Target Site Finding
Source: PLoS One. 2014 Oct 21;9(10):e108575. doi: 10.1371/journal.pone.0108575 (PMC4204827; doi:10.1371/journal.pone.0108575)
Supplement: Table S1 — Summary of the simulation parameters used. (PDF) [file pone.0108575.s003.pdf]

Table S1: Simulation file parameters

Quantity	Unit	Fig3BC	Fig3D	Fig4	Fig5A	Fig5CD (default)
number of DNA filaments	number	10	10	20	1 for 1D, 10 for IST	50
DNA length	nm	2.5 to 400	2.5 to 400	310	1400 for 1D, 140 for IST	1000
DNA width	nm	2.6	2.6	2.6	2.6	2.6
3D diff. coeff.	$\mu m^2/s$	2.72	2.72	2.72	2.72	2.72
3D rms step length	nm	23.3	23.3	2.33	2.33	23.3
1D diff. coeff.	$\mu m^2/s$	0.0262	0.0262	0	0.0262	0.0262
1D rms step length	nm	2.29	2.29	0	0.229	2.29
adsorption coeff., k_{on}	µm/s	1.7	1.7	1850	0	10
adsorption probability per time step	unitless	0.0181	0.0181	1	0	0.103
desorption coeff., k_{off}	s^{-1}	11.6	0.0242 to 1160	2120	0	11.6
desorption probability per time step	unitless	0.00115	1.15e-6 to 0.109	0.00116	0	0.00111
TF-TG association rate, k_{assoc}	$M^{-1}s^{-1}$	100000	100000	not computed	100000	100000
binding radius	nm	1.72	1.72	4.5	0.433	2.01
TF-TG dissociation rate, k_{diss}	s^{-1}	0.025	0.025	0	2.89e-12	2.89e-18
dissociation probability per time step	unitless	2.50e-6	2.50e-6	0	2.89e-15	0
reaction permissions		front+front	front+front	front+front	front+all	front+all
IST transfer rate constant	s^{-1}	0	0	0	0 for 1D, 1000.5 for IST	117
simulation time, t_{max}		1 hr	30 min	1 min	100 s	30 min
time step	ms	0.1	0.1	0.001	0.001	0.1

White backgrounds represent values that were entered into simulations; green backgrounds represent values that were computed by Smoldyn. Fig2A and Fig5B are as Fig5CD, apart from DNA dimensions, for ease of visualisation.
