# Supplementary material for: An Integrated Model of Transcription Factor Diffusion Shows the Importance of Intersegmental Transfer and Quaternary Protein Structure for Target Site Finding
Source: PLoS One. 2014 Oct 21;9(10):e108575. doi: 10.1371/journal.pone.0108575 (PMC4204827; doi:10.1371/journal.pone.0108575)
Supplement: Code S1 — Archive of Smoldyn configuration files, Matlab scripts and Python code for simulations and data analysis. See enclosed README file for details. (ZIP) [file pone.0108575.s004.zip › Code S1/README.rtf]

Code for figures shown in Schmidt H, Sewitz S, Andrews SS & Lipkow K, PLoS ONE 2014.Smoldyn and detailed documentation can be downloaded from www.smoldyn.org.For Smoldyn simulations, - open a Terminal application, - cd into the "SmoldynFiles/FigXX" folder, with XX being the number of the figure in the paper, and, if applicable, into one of the subfolders- type:   smoldyn sld0.txt- if a graphics window appears (Fig2A and Fig5D), you can now adjust the size of the window by dragging on one corner of the window, and the size of the nucleus by pressing the '=' or '-' keys and orientation of the simulated nucleus by pressing the arrow, or 'x', 'y', 'z' keys; then start the simulation by pressing the space bar.The simulations will create one or more output files. Analyse these with the m-files found in the MatlabFiles folder.Adobe Illustrator was subsequently used to improve the colourscheme and appearance of some plots.Most simulations were submitted to a cluster using Condor, with customised submit scripts. Some modification of the m-files might be required if you run fewer repeats, or use a different naming system for the simulation output files.For Fig5CD, the colour code is the following:magenta: ISTa1Dgg3Dred: ISTa1Dorange: 3De1DeISTyellow: 3De1Dgreen: 3Dgg1Dblue: 3De1Dpurple: 3Donlygrey: 1Donlyblack: ISTonlyfig6.py is the Python script used to extract oligomer numbers from the non-redundant 3D protein complex database.
